# Investigating the challenges of biogas provision in water limited environments through laboratory scale biodigesters

**DOI:** 10.1080/14786451.2023.2235022

**Published:** 2023-07-21

**Authors:** Jennifer Wardle, Davide Dionisi, Jo Smith

**Affiliations:** a School of Biological Sciences, University of Aberdeen, Aberdeen, UK; b School of Engineering, University of Aberdeen, Aberdeen, UK

**Keywords:** Biogas, household-scale anaerobic digestion, water demand, sub-Saharan Africa, organic resource use

## Abstract

The potential for biogas provision through household-scale anaerobic digestion in rural sub-Saharan Africa is limited due to perceived water shortages. The most common substrate is animal dung diluted 1:1 with water. Two experimental methods tested the potential of reducing water demand. The first experiment compared the chemical oxygen demand (COD) and volatile solid removal of four cow dung dilutions ranging from 3.5-10.6% total solids. In the second experiment, bioslurry filtrate was recirculated back into the fresh substrate at different concentrations. The highest COD removal rate of 28.3% was obtained from mixing equal volumes of dung with filtrate (mean total solids 7.4%) while the highest methane production rate of 0.40 g/L/day, calculated from COD balance, was obtained from undiluted cow dung (total solids 10.6%). Results suggest the potential for a 75-100% reduction in water demand.

## 1 Introduction

### 1.1 Energy poverty in sub-Saharan Africa

Access to energy for basic household cooking requirements remains problematic for millions of people in low-income countries (Surendra et al. 2014). In sub-Saharan Africa (SSA), only 22% of the rural population has access to electricity (Nyiwul 2020). Not only is energy in short supply, but available sources are often inefficient and harmful to human health and the environment (Guta et al. 2017). Respiratory tract infections linked to solid fuel combustion account for 3.5 million premature deaths per annum, predominantly in South Asia and in SSA (IEA 2016). Electrification by centralised national grids is not a feasible option in many areas of SSA due to the low population density of rural dwellers, meaning decentralised energy solutions are more appropriate (Quak 2018). Decentralisation of energy supply can aid empowerment of rural households and create resilience against fluctuating prices and the eventual decline of fossil fuels (Dionisi et al. 2018; Guta et al. 2017).

Biogas from small-scale anaerobic digestion (AD) has been proposed as a clean source of renewable energy for subsistence farmers in SSA. Unlike with industrial processes where the production of by-products may present a waste disposal problem (Saady and Massé 2015a), in SSA, the bioslurry by-product has high potential to increase food security, especially on subsistence farms (Uckert et al. 2017) which suffer from soil degradation and where chemical fertilisers are often unaffordable. Moreover, the energy conversion efficiency for biogas is 55% compared to <12% for wood and dung combustion on open fires (Chica and Pérez 2019; Kamp and Bermúdez Forn 2016) meaning less fuel is required. Unlike other off-grid systems, such as solar energy, biogas has the advantage of being easily stored without the need for batteries (Kemausuor, Adaramola, and Morken 2018).

The majority of work conducted to improve the efficiency of AD applies to large-scale industrial batch systems and co-digested materials. Even in such conditions, AD of dung with high solid-content remains understudied (Saady and Massé 2016). As biogas yield is enhanced by co-digestion which improves the C:N ratio and mixes labile and recalcitrant feedstocks, it has been suggested that crop residues should be mixed with dung (Tucho et al. 2016). In rural SSA, co-digestion with materials beyond dung is often infeasible due to low availability of machinery to grind high cellulose material into a size that is degradable and releases intracellular organic matter (Rocamora et al. 2020). Semi-continuous digestion is necessary for households to maintain a continuous biogas supply and enables use of fresh dung before it begins to dry out and degrade. However, there is little research on the efficiency of small, low-tech, dung-fed, semi-continuous AD in resource poor settings, with much of the existing literature focusing on physical barriers, socio-economic aspects of uptake or broader potential benefits to human health and soil productivity (Kelebe et al. 2017; Kemausuor, Adaramola, and Morken 2018; Orskov et al. 2014; Tolessa, Louw, and Goosen 2022). This is an important oversight as AD could provide a relatively high proportion of the energy demand of SSA; for example, in Nigeria, 18-36% of the annual energy consumption of 4.88 EJ could be provided by AD if manure, agricultural residues and the organic fraction of municipal solid waste were used efficiently (Dionisi et al. 2018).

### 1.2 Water availability

A key limitation to AD in SSA is the availability of water (Bansal, Tumwesige, and Smith 2017; Roopnarain and Adeleke 2017), particularly in dry season (Hewitt et al. 2022; Wardle et al. 2021), with typical recommendations being that cow dung should be diluted with water at a 1:1 ratio (Bansal, Tumwesige, and Smith 2017; Sime 2020; ter Heegde 2010) while 40% of the population of SSA suffer from water scarcity (Rupf et al. 2017). Water collection is labour intensive with average national weekly collection times in rural areas reported to be 4.5–6.5 h per week in Ethiopia and Kenya (Caruso 2017; Cassivi, Waygood, and Dorea 2018). Use of AD should avoid increasing the time and labour input due to the requirement to obtain additional water. While water harvesting during the wet season could provide relief on water collection, it cannot provide enough for the currently recommended dilution rates (Bansal, Tumwesige, and Smith 2017) and is not available throughout the year. Failure of anaerobic digester projects in SSA is widespread, yet heavily under-investigated due to a taboo of addressing failures (Kalina, Ogwang, and Tilley 2022), although water shortages and inability of users to mix the correct dung to water ratios are certainly contributary factors (Hewitt et al. 2022).

To increase the reach of AD in SSA, it is necessary to reduce the water demand. Options are to reduce the dilution rate or to recycle the liquid component of the digestate (Smith et al. 2013). Recirculation of liquid digestate percolated through filters is common practice in batch systems where it can aid mixing and increase exposure of methanogens to nutrients (Rocamora et al. 2020). However, liquid recirculation is not typically used in continuous systems as it can lead to accumulation of inhibitory substances such as volatile fatty acids (VFA) (Pezzolla et al. 2017); which frequently causes system failure (Meegoda et al. 2018).

Increasing the solids content in AD has been explored in industrial settings to facilitate smaller digester sizes, saving costs on construction and operation (Saady and Massé 2015b), but is not common practice in resource poor settings. Other than being more suitable for precipitation limited conditions, a higher solids content results in drier digestate which is easier to handle (Ahn et al. 2010), and can potentially produce a higher methane yield per kg of substrate. Current dilution recommendations may be due to the potential accumulation of VFA or other inhibitory compounds, such as ammoniacal nitrogen (N) (ammonium plus free ammonia) when the feeding rate is too high (Rocamora et al. 2020). However, when co-digesting dung with food waste in batch systems, Wang et al. (2020) found no significant difference between methane yield from 5–15% total solids (TS), but a reduction at 20% TS, which is similar to Luo et al. (2021) who found a degree of inefficiency with 17.5% TS, but a greater efficiency at 15% TS compared to 12.5% TS. Moreover, Saady and Massé (2015a) even observed successful AD of dung and straw up to 35% TS, followed by a successful 35% reduction of VS with the same feedstock at 27% TS in a 21 d cycle (Saady and Massé 2016). The authors found the TS of cattle dung from six households in Ethiopia to be 18.9% (standard error (SE) 1.1) TS in dry season, with a similar 18.4% TS reported by Budiyono, Johari, and Sunarso (2010). This suggests 1:1 dilution may be in excess of requirements (only ~9.5% TS) and may therefore be hindering the development of sustainable energy implementation in much of SSA.

Here two experiments were conducted in order to assess whether changing current AD practices could increase access to biogas for rural households. First, was an investigation into the effects of reducing the quantity of water used in the dilution process which currently stands at a minimum of 1:1 dung to water. This is achieved by adjusting the organic loading rate (OLR) of dung-only systems. An alternative water-saving strategy was then investigated, which entailed recycling the water from the digestate using simple methods that can be replicated in low-income conditions, while monitoring the VFA content of the reactors.

## 2 Materials and methods

### 2.1 Analytical methods

Approximately 18 litres of fresh cow dung from a farm in Aberdeenshire was homogenised with an electric blender then kept frozen at -20°C until processed. The TS content was obtained by drying 10 samples of pre-weighed homogenised dung at 105°C overnight and reweighing. The volatile solids (VS) content was measured by combustion of the dried solids in a furnace at 550°C for 2 h (Meegoda et al. 2018). The chemical oxygen demand (COD) of the dung samples was measured using a Spectroquant Nova 60 photometer (Merck 2019). Spectroquant 5000–90000 mg/L COD test kits with dung/digestate mixed at 1:4 with water were heated at 148°C in a thermo-reactor for 120 min and left to cool, with swirling after 10 min, before COD levels were detected. The difference between the COD of the substrate and digestate was used as a proxy measure for methane production throughout. As fermentation products such as acids and alcohols are accounted for in the COD of the digestate, and the carbon dioxide in the biogas is already fully oxidised, COD is a reliable measure of methane production alone, with the two measures found to be highly consistent when measured separately (Dumas et al. 2010). The pH and temperature were measured on every feeding day throughout both experiments. After drying out dung at 50°C and milling for 2 min, 6 samples were sent for C:N analysis on a Thermo Finnigan Flash EA1112 Elemental Analyser using a flash combustion and gas chromatograph method. The means of the 3 experimental replicates were used for all analysis and means of 3 technical replicates were also used for VFA analysis. For gas chromatography preparation, ten dilutions with distilled water of SIGMA SUPELCO volatile free acid mix with a concentration of 10 mM were used to create a standard curve in a Thermo Scientific Trace 1300 gas chromatograph for liquid samples. Settings were as described by Silva and Dionisi (2020). For all 9 VFA calibrated, the r^2^ was at least 0.996.

The mean density of the dung was established as 95.2 g per 100 mL and the volume of dung was thereafter measured by weight due to fibres causing inaccuracy in measuring quantity by volume for small volumes of manure. The TS of the dung were 10.6% w/w (SE 0.09), the VS were 80.6% (SE 0.10) of the TS and the COD was 190.8 g/L (SE 9.01). The mean C:N ratio of the dung was 12.77:1 by weight with a mean N content of 3.34% (SE 0.03) and C content of 42.61% (SE 0.10) (percentages refer to the dry weight).

### 2.2 Dilution experiment

**Figure 1:**
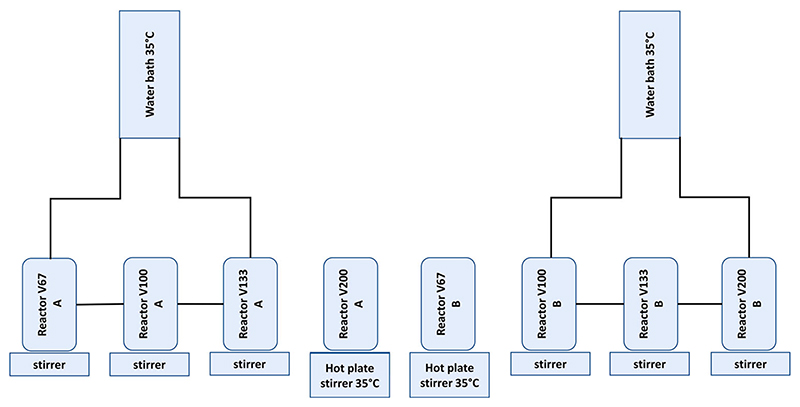
Set up of first two replicates of the dilution experiment.

Eight continuous stirred tank reactors with a semi-continuous feed, working volume of 200 mL and a retention time of 25 days were maintained at 35°C for a period of 75–78 days after the first feeding event between May and December 2018 (reactors A and B). A mix of hot plates and water baths was used after piloting set-ups for the most consistent temperatures. This consisted of 2 replicates for each OLR (Figure 1) and was followed by a third replicate running from December 2018–March 2019 (reactors C). Digester operating conditions are shown in Table 1. The digesters were inoculated with a total volume of 200 mL, made of dung and deionised water in the same proportions as for the feed of each reactor plus 2 g of dry, mineral garden soil without chemical additions was added to each reactor as an additional inoculum. Reactor groups of three replicates each will be referred to by the initial volume of dung in the reactors shown in Table 1, i.e. V is volume, V200-200 mL undiluted dung (TS 10.6%), V133–133 mL dung and 67 mL water, V100–100 mL dung and 100 mL water, V67–67 mL dung and 133 mL water.

Substrate was fed into the digesters via rubber piping in the lids 3 times per week using preloaded 20 mL syringes which were thawed from frozen. Samples were taken from digesters using a syringe before fresh substrate was added for measurements of TS, VS and COD. Digesters were then flushed with 75 mL of N gas to eliminate oxygen from the gas collection chamber prior to access holes in the lid being replugged.

### 2.3 Water separation and recycled liquid digestate experiment

Six continuous stirred, semi-continuous feed tank reactors were set up at 35°C and started up with 100 mL cow dung and 100 mL water, as 1:1 dilution is the most commonly used ratio for household digesters in SSA. The feed into the reactors consisted of 50% cow dung in all cases. The remaining 50% of the feed was either a 50:50 mix of recycled liquid digestate and deionised water (RLD50) or entirely made of recycled liquid digestate (RLD100). All digestate was filtered through a 1 mm sieve. A simple sieving method was used as it can be replicated in SSA. Recycled liquid digestate for the first 25 days was obtained from V100 reactors from the dilution experiment (i.e. recycled once), for the next two 25 d retention periods it was obtained from the digesters in use (i.e. recycled twice then three times). V100 reactors were used for obtaining filtrate as they had the same 1:1 dung to water dilution rate. Each reactor received the same 2 g of dry, mineral garden soil as additional inoculum. Temperature and pH inside the reactors were measured at every feeding interval which was 3 x per week. Samples for COD, gas chromatography, TS and VS were taken a minimum of once per week. The filtered water was also analysed to compare properties in the different phases, i.e. after being recirculated once, twice and three times.

### 2.4 Calculations and statistical methods used

In the R software environment, the plot(lm) function and Levene’s Test were applied to check that assumptions were met for the analysis of variance test (ANOVA) for differences between reactor groups. The non-parametric Kruskal Wallis test was applied instead of ANOVA if assumptions of normality or variance were not met.

The percentage reduction of COD, *P*_COD_R__ (%), was calculated using Equation 1, where *COD*_s_ is the theoretical COD in the feed (g/L), based on untreated dung adjusted for dilution, and *COD*_d_ is the COD of the digestate (g/L). The same method was applied to calculate the percentage reduction in VS (Equation 2), where *VS*_s_ and *VS*_d_ are the VS in feed and digestate respectively (g/L). The rate of COD removal, *R_COD_*_R_ (g/L/day), was calculated using Equation 3, where *t*_RT_ is the retention time (25 days). The rate of methane production, *R*_CH4_ (g/L/day), was then calculated from *R_COD_*_R_ by using the COD balance and assuming the ratio of methane to COD removal is 1:4, which derives from the stoichiometry of the complete oxidation of methane (Harb et al. 2015), as shown in Equation 4. Equation 5 shows the COD reduction over the digestion period from the dung produced each day by one cow, *R*_COD_R,cow__ (kg/cow/day). In using Equation 5, the dung production (*M*_dung_*cow*__) was assumed to be 14 kg per cow per day (a conservative estimate obtained from the 10 kg per day suggested by (Kabera et al. 2016) and 16–20 kg per day suggested by (Gupta, Aneja, and Rana 2016), and the density of dung (*d*_dung_) was assumed to be 952 g/L). Similarly, the equivalent CH_4_ production of dung per cow per day, *R*_CH4,cow_ (kg) was calculated from *R*_COD_R,cow__ (equation 6) by using the conversion factor 1:4 between methane and COD, as discussed above. Finally, *R*_CH4,cow_ was multiplied by the heating value (HV) of CH_4_ which is 55.00 MJ/kg (World Nuclear Association 2020) to establish energy output per cow, E (MJ/cow/day), Equation 7.

(1) $$P_{{\text{COD}}_{\text{R}}}=\left(\frac{COD_{\text{s}}-COD_{\text{d}}}{COD_{\text{s}}}\right)\times 100$$

(2) $$P_{{\text{VS}}_{\text{R}}}=\left(\frac{VS_{\text{s}}-VS_{\text{d}}}{VS_{\text{s}}}\right)\times 100$$

(3) $$R_{{\text{COD}}_{\text{R}}}=\frac{P_{{\text{COD}}_{\text{R}}}\times COD_{\text{s}}}{100\times t_{\text{RT}}}$$

(4) $$R_{\text{CH}4}=\frac{R_{{\text{COD}}_{\text{R}}}}{4}$$

(5) $$R_{{\text{COD}}_{\text{R},\text{ cow }}}=\frac{P_{{\text{COD}}_{\text{R}}}}{100}\times COD_{\text{s}}\times \frac{M_{{\text{dung}}_{\text{cow }}}}{d_{\text{dung}}}$$

(6) $$R_{\text{CH}4\text{, cow }}=\frac{R_{{\text{COD}}_{\text{R},\text{ cow }}}}{4}$$

(7) $$E=R_{\text{CH}4\text{, cow }}\times HV$$

## 3 Results

### 3.1 Effect of different dilution rates on COD and VS reduction

**Figure 2:**
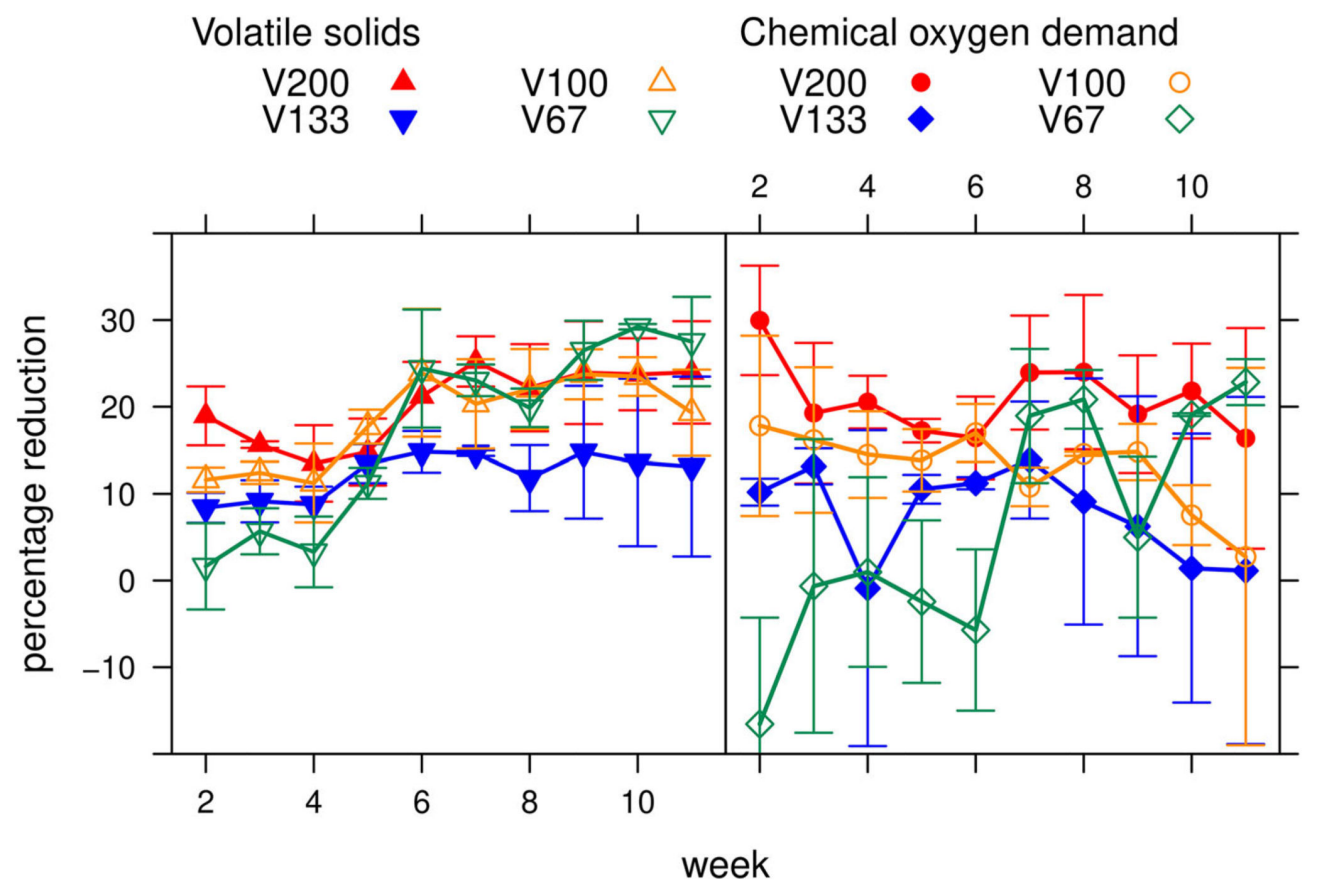
Reduction of chemical oxygen demand (*P*_COD_R__) and volatile solids (*P*_VS_R__) (%) over time. Error bar = standard error.

All dilution rates exhibited a reduction of COD and VS with Figure 2 showing the mean values for the three replicates of each treatment. Some problems were encountered with the replicate treatments; reactor C for treatment V133 failed at approximately day 40 and did not recover, while reactor C for treatment V67 had an initial accumulation of COD and VS in the first half of the experiment but recovered around day 40. (See Supplementary Material for individual reactors).

**Figure 3:**
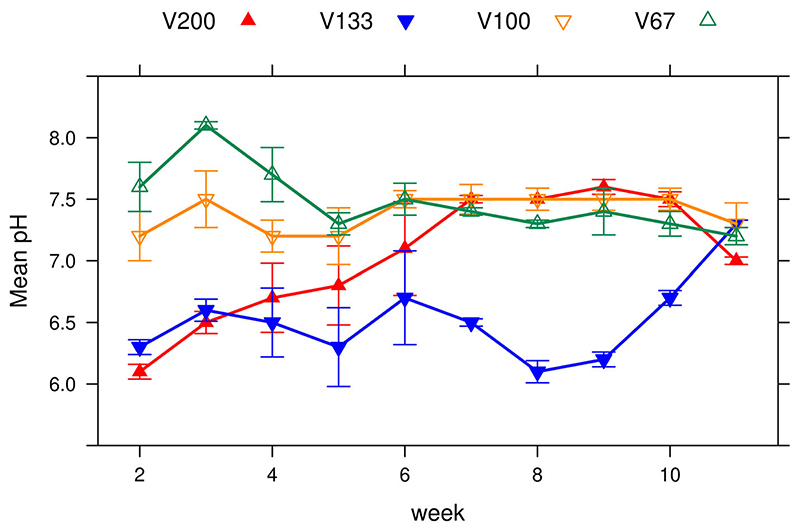
Mean values of pH over running time. Error bar = standard error.

In reactor group V200 there is a steady increase in pH from a starting point of pH 6.1 to a maximum of pH 7.6 at days 40–60 (Figure 3). This is associated with higher VS reduction rates occurring in the early digestion process seen in Figure 2, occurring when the pH in those reactors was at its lowest. This trend was not followed in other reactors. All reactor groups except for group V133 stabilised at around week 6 with the range being narrower from that point onwards. Group V100 has the most consistent pH over time with a range from pH 7.2–7.5. The maximum range of pH was observed in group V200, although group V133 was the most inconsistent with large fluctuations between measuring points and the lowest median value of 6.5 compared to 7.0–7.5 in the other groups.

A Kruskal Wallis test found a significant difference between the percentage COD reduction of the different treatments (*p* = 0.0005). Mean data from across the entire running time indicates that treatment V200 (undiluted dung at 10.6% TS) had the highest COD removal efficiency (*P*_COD_R__) at 20.9% (Table 2). The low rate for reactor group V133 was partially due to the failure of V133 C. If V133 C is eliminated from the analysis, the mean COD removal for group V133 is increased to 13.4% but the difference between groups remains statistically significant (*p* = 0.0008). For reactor group V67, the standard error in the mean COD reduction is particularly high, indicating significant uncertainty in the measurement of COD reduction in this case. As shown in Table 2, reactor group V200 also performed better in terms of VS reduction over the whole running time. Reactor group V133 yielded the lowest VS reduction rates, again, partially due to the failure of V133 C. Most reactors appear to perform better when measured by VS reduction rather than COD reduction; this will be discussed further below. When assessing the percentage reduction of COD compared to VS in the most effective reactors, i.e. V200, there is no significant difference between the means of the two (*p* = 0.77) over the running period.

The daily removal rates of COD shown in Table 2 correspond to a CH_4_ production rate (*R*_CH4_) of 0.40, 0.10, 0.12 and 0.05 g/L/day for groups V200, V133, V100 and V67 respectively. The methane production rate calculated from the COD removal can be compared, with some assumptions, with the total biogas production (CO_2_ + CH_4_) which can be estimated from the VS reduction. Indeed, the VS reduction corresponds to the conversion of the organic matter into components in the gas phase (mainly CO_2_, CH_4_) and into inorganic components in the liquid phase (mainly H_2_O). With the assumptions of ignoring water production and of assuming that all the produced CO_2_ goes in the gas phase (in reality some of the produced CO2 will dissolve into water), the VS reduction from Table 2 corresponds to the total production of gas (CO_2_ + CH_4_). From Table 2 and from the OLR_VS_ in Table 1, we can estimate a total gas production of 0.66, 0.26, 0.30 and 0.19 g/L/d for groups V200, V133, V100 and V67 respectively. The total gas production estimated from the VS removal is higher than the methane production calculated from the COD removal, which is as expected considering that VS removal also includes the carbon dioxide production. The slight fluctuations of COD and VS removal from the reactors indicates a steady state has been reached at an early stage.

### 3.2 Recycled liquid digestate experiment

The properties of the feeds RLD100 and RLD50 used in the experiments with recycled filtered digestate, after being digested once, twice and 3 times, are shown in Table 3. For the RLD100 reactors, the mean OLR of VS was 2.14 kg/m^3^/day and the mean COD was 122.59 g/L. For the RLD50 reactors the mean OLR based on the VS was 1.84 kg/m^3^/day with the mean COD being 108.99 g/L. Properties can be compared to those for given for V100 in Table 1 which has 1:1 dung: deionised water ratio.

#### 3.2.1 Chemical oxygen demand and volatile solid reduction with recycled filtrate

**Figure 4:**
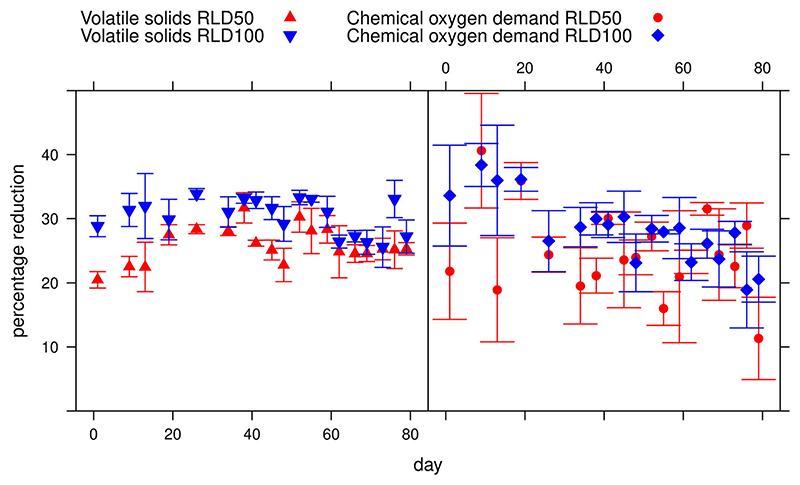
Reduction of chemical oxygen demand (*P*_COD_R__) and volatile solids (*P*_VS_R__) (%) over time with liquid digestate recycling. Error bars = standard error.

When considering reduction of COD across the whole running time, a Kruskal Wallis test found no significant difference (*p* > 0.05) between reactor groups, although group RLD100 which used 100% recycled liquid input performed slightly better than group RLD50 with 50% recycled liquid input (Table 4). The VS reduction, however, was significantly different between groups (p < 0.001) which may be explained by higher levels of accuracy in mass measurement used for VS than volumetric measurements for COD. Again, for the most effective reactors, i.e. RLD100s, there is no significant difference between percentage COD and VS reduction (*p* = 0.22) (Figure 4).

Kruskal Wallis analysis was undertaken to check for differences between the three liquid substrate recycling phases, i.e. 1 = days 0–25 recycled once, 2 = days 26–50 recycled twice and 3 = days 50–78 recycled three times (Table 4).

#### 3.2.2 Volatile fatty acids over running time

**Figure 5:**
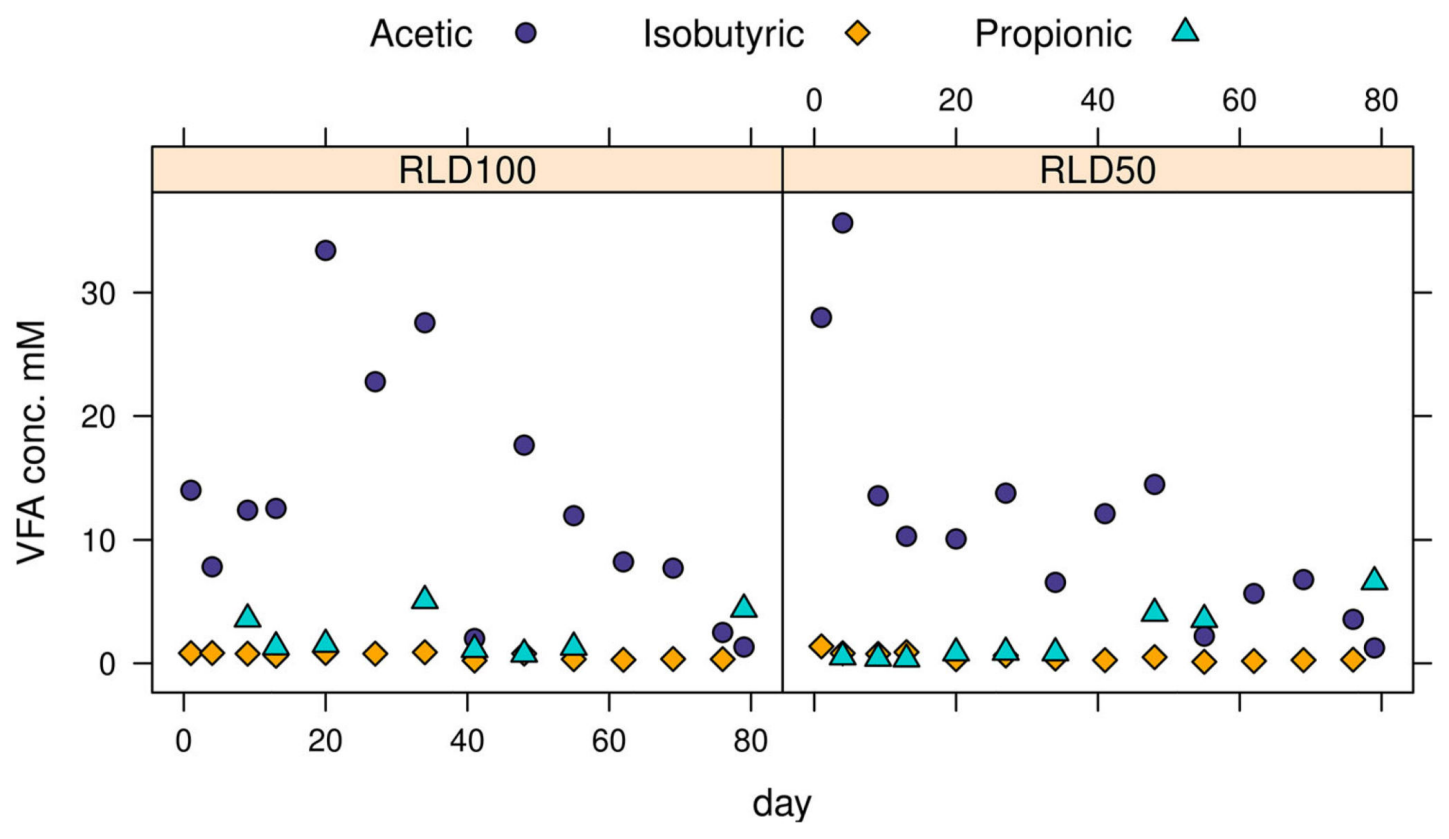
Mean volatile fatty acid levels (VFA) over running time of reactors.

The VFA with the greatest abundance in all of the reactors was acetic acid, followed by propionic acid (Figure 5) which were found at maximum concentrations up to 2.1 and 0.5 g/L respectively. Most of the other VFA were found infrequently at low levels with the maximum concentrations of hexanoic, isocaproic, n-Heptanoic and valeric acids being only 0.13, 0.09, 0.23 and 0.26 mM or 14.7, 10.1, 29.9 and 26.2 mg/L respectively. Only the VFAs with the top three concentrations are shown in Figure 5 to avoid obscurity from overlaps as most are close to zero. No significant difference in any individual VFA levels were found between treatment groups RLD50 and RLD100 with 50% and 100% recycled liquid digestate. The quantity of acetic acid detected in the samples decreased over time (Figure 5). Quantity differences of total VFA between the three phases within each group were not significant but the lowest quantities were found in phase 3 for both.

There was no clear association between VFA and pH, with pH fluctuating over time. Reactor group RLD50 had a mean, minimum and maximum pH of 7.5, 7.3 and 7.8 while group RLD100 had the corresponding figures of pH 7.6, 7.4 and 7.8.

### 3.3 Methane production

In the dilution experiments, the highest COD removal (*P*_COD_R__ 20.9% as average value) was observed when no water was added. Since the highest COD removal rate (*R*_COD_R__) was 1.59 g/L/day, the highest observed COD removal efficiency corresponds to an estimated methane production (*R*_CH4_) of 0.40 g/L/day.

In the diluted reactors, the COD removal efficiency was in the range 6.2–13.0%, which corresponds to an estimated methane production in the range 0.04–0.12 g/L/day. This comparison clearly shows the benefit of using no dilution to increase the methane production rate per unit volume of reactor, although there was a high degree of error in COD measurements (Figure 4). Assuming a daily dung production rate per cow to be 14 kg or 14.7 L, 20.9% COD removal corresponds to a methane production (*R*_CH4,cow_) of 146.4 g methane per cow per day (See Supplementary Material). As the enthalpy of methane combustion is considered to be 55.00 MJ/kg, the daily energy potential provided by one cow (E) is 8.1 MJ which equates to 2,938 MJ per year. As the combustion efficiency of a biogas stoves is 55% (Kamp and Bermúdez Forn 2016), each cow provides 1,616 MJ of useful cooking energy per year.

In the recycling experiments, the highest mean COD removal was 28.26% which occurred in the RLD100 group where all of the liquid added to the dung was recycled filtrate. In this case the COD removal was 1.39 g/L/day corresponding methane production of 0.35 g/L/day. Assuming the same dung production rate per cow and an initial COD of 122.59 g/L, this corresponds to a production of 127.4 g methane per cow per day, providing 6.9 MJ of energy per cow each day, equating to 2,557 MJ per year, or 1406 MJ of useful cooking energy.

## 4 Discussion

The best performance in terms of percentage COD and VS reduction in the dilution experiments was found in reactor group V200 containing undiluted cow dung (TS 10.6%) with a mean COD reduction of 20.87% and VS reduction of 20.31% when considering all days across the running time. As the TS content of this manure was 10.6%, this provides similar findings to Budiyono, Johari, and Sunarso (2010), who found that a TS content of 9.2% gave the highest biogas yield. However, it should be noted that there is a large difference in the reported TS content of cow dung, ranging from 11% (Rupf et al. 2017), to 12–14% (Saady and Massé 2015b) to as high as 28.7% (Haryanto et al. 2018), with many studies not reporting the figure at all. The high variability of the TS content of cow dung can attributed to different feeding practices between farms (Møller, Sommer, and Ahring 2004), but may also be due to evaporation before collection and the content of bedding material. Furthermore, seasonal differences are observed within the TS content of cattle dung of the same animals according to whether it is wet or dry season (Wardle et al. 2021). It is therefore difficult to establish a ‘one-size-fits-all’ practice in terms of dung dilution and digester design. This is problematic as a known cause of failure in improved cookstove projects is lack of uniformity in design, which increases training difficulties for users and installers (Troncoso et al. 2011). In this study the TS and VS have been reported throughout so appropriate modifications can be made to dilution rates.

Over the whole period of the recycling experiment, the most productive reactors were in group RLD100 which used 50% wet manure with the whole of the additional liquid input obtained from filtered digestate. The mean reduction in COD was 28.6% and in VS was 29.9%. Both reactor groups in the recycling experiment performed better in terms of percentage COD and VS reduction than their 50:50 dung: water counterpart, Group V100, in the dilution experiment which had a VS reduction of 20.3%. The reactors appear to have benefitted from the recycling of the filtrate.

Contrary to VFA build-up in the filtrate, the pH of the filtrate was alkaline, probably due to the low C:N ratio of the substrate (C:N = 12.8). Although optimum C:N ratios for degradations are usually quoted as 20–30, Guarino et al. (2016) tested a wide range of C:N ratios between 9.8–50.1 and found that the sample with the C:N ratio of only 9.8 had the highest rate of methane production. This suggests that co-digestion to increase the C:N ratio may not be necessary. The highest concentrations of VFA occurred in the early period of running the digester and gradually decreased; this is similar to results found by other authors (Dong, Zhenhong, and Yongming 2010; Saady and Massé 2016). This may be due to acidogenic bacteria having a faster growth rate than acetogens and methanogens (Meegoda et al. 2018; Pezzolla et al. 2017; Yin et al. 2018) meaning VFA producers were first to take dominance but soon became balanced by microbes from the succeeding groups, with hydrolysis potentially becoming the rate limiting step (Dong, Zhenhong, and Yongming 2010). Accumulation of total ammoniacal-N is less likely to cause inhibition than VFA accumulation due to a buffer system where methanogens, the most sensitive organisms in the system, are the first to be killed by a high pH, allowing for increased levels of VFA to neutralise the conditions (Dong, Zhenhong, and Yongming 2010; Rocamora et al. 2020). Despite ammoniacal-N levels being an important parameter for the success of anaerobic digestion, inhibitory levels are difficult to quantify as it is dependent on pH, temperature, and microbial communities within each digester, although levels of 1000–1500 mg/L are often associated with system inhibition (Capson-Tojo et al. 2020). Throughout the recycling experiment, the pH of the individual reactors ranged between pH 6.7–7.9 in group RLD50 and pH 7.0–8.0 for group RLD100, with respective mean values for each treatment group being pH 7.5–7.8 and pH 7.6–7.8, conditions therefore being favourable for methanogens which are neutrophiles (Budiyono, Johari, and Sunarso 2010; Rocamora et al. 2020). Future work investigating the potential of using filtrate from further recycling phases would benefit from the specific monitoring of ammoniacal-N levels, especially as the C:N ratio of the dung used, at 12.77:1, was relatively low compared to other figures quoted in literature, with higher nitrogen levels potentially being problematic.

Our results suggest that 1406 MJ of useful energy is produced per cow per year with 100% filtrate recycling (RLD100), while 1,616 MJ of useful energy is produced per cow per year with no dilution of dung (V200). The quantity of dung per head of cattle differs between sources, with many referring to that of industrialised systems in high-income countries. It is suggested that 6 GJ of useful energy is required annually for cooking by typical family of 6 in rural areas of developing countries (Tucho et al. 2016). Our results suggest that this annual energy requirement would need 4 head of cattle, producing 6.5 GJ using undiluted dung at 10.6% TS (V200), or 5.6 GJ could be achieved using filtrate recycling at 7.4% TS with 4 head of cattle (RLD100). The daily dung production rates used were modest to compensate for a 100% collection rate of fresh dung being unlikely. The figures therefore have some credence with the recommendations for a minimum of four cows to be used for household biogas production in Ethiopia (Kamp and Bermúdez Forn 2016) and for 8 m^3^ digesters in Rwanda (Kabera et al. 2016). It appears, however, that for smaller families and/ or smaller digesters, 2–3 head of cattle may suffice.

It was found that undiluted dung (V200) had the largest energy potential due to the higher concentration of volatile solids while the filtrate dilution (RLD100) had the more efficient COD reduction. Whether it is preferable to implement AD using diluted mixtures, so maximising the efficiency of feedstock digestion, or to implement AD using more concentrated mixtures, so maximising the energy production with lower input of water, depends on whether it is the dung or the water that is most limiting. If water availability is the most limiting factor, then this work highlights the potential to increase the concentration of the feedstock or recycle water, so reducing the demands on water while still producing sufficient energy for the household. While it is beneficial to have a plentiful supply of biogas to prevent or minimise the practice of fuel stacking (maintaining the use of dirtier, less efficient fuel sources in conjunction with improved sources), excessive biogas production is undesirable as it can lead to households releasing unused gas into the atmosphere to reduce accumulating pressure (Bruun et al. 2014). Since methane is a strong greenhouse gas, 28 times more potent than carbon dioxide over a 100 year period (IPCC 2019), this has negative impacts with regards to climate change.

The results of the dilution ratio experiment suggest that the current practice of 1:1 dung dilution is not necessary and may be severely limiting the potential of low-tech AD in SSA. A change of reactor design to allow a wider input pipe diameter could facilitate an increase in substrate viscosity to allow undiluted dung input. A higher dry matter content also reduces separation and scum formation in digesters compared to systems which have a higher liquid input (Edelmann and Engeli 2015; Kothari et al. 2014; Rajendran, Aslanzadeh, and Taherzadeh 2012) and could therefore reduce the number of technical problems encountered. Increasing the TS content of substrate would have the additional benefit of reducing construction costs of future installations as the required size would be reduced.

Positive results were also found with recycling liquid digestate. The success of continued recycling means that fresh water inputs are not required every time a new substrate is added, although some additions may be required to compensate for losses by evaporation. As water was recycled back to the reactor over 4 × 25 d retention times, which may be greater in ambient temperature digesters, the same water could theoretically be maintained over the peak dry season in at least some parts of SSA, with rainwater harvesting or other available sources being used for water renewal thereafter. It was found that water consumption could be reduced by at least 75% with a positive impact on methane production. For the RLD system, the digestate separation could be achieved through a simple hessian filter, which would also benefit small-holder farmers by reducing the volume of digestate to be transferred to the soil as a conditioner and fertiliser. Both systems would avoid the direct combustion of fuelwood and dung, thereby reducing deforestation and the harmful impacts to human respiratory health, while benefiting the nutrient and C content of the soil.

As can be seen in Figures 2 and 4, the fluctuation in COD reduction is much greater than for VS reduction. This is most likely to be due to the relatively large error margins involved in working with a fibrous substance in small volumes. Although COD is often considered a more reliable measure than VS (Meegoda et al. 2018) and consistent with methane production (Dumas et al. 2010), samples for COD analysis had to be measured by volume whereas for VS it was measured by mass, giving a more consistent result. Future study would benefit from additional gas measurements, allowing direct measures of methane production to corroborate percentage COD reductions. In this case there was no significant difference between the percentage COD and VS reductions in the most efficient reactor in each experiment and final percentage reductions were very similar, i.e. V200 had a 20.9% reduction of COD compared to 20.3% for VS, while RLD100 has a 28.3% reduction of COD compared to 29.9% for VS. The highest degree of COD error was observed in reactor group V67, which also has the greatest degree of VS error, as shown in Table 2. The range of mean percentage reduction values was greatest in this group for both COD and VS reduction with values between –16.56% and 22.84% for COD and 1.64% and 29.23% for VS. The reasons for the overall bad performance of COD measurements in this group is unclear, but is partially attributable to the particularly bad performance in reactor V67 replicate C (Supplementary Material). Another limitation of the study is that filtrate was only recycled three times. Further study is required to test how many times the filtrate can be recycled without negative impacts on methane production, as well as scaling-up the reactor sizes and dung quantities involved. While this degree of recycling is sufficient to increase access to biogas energy by reducing the number of water collection trips by 75%, the potential for further reduction is still unknown.

## 5 Conclusion

The findings have promising implications with regards to increasing the potential use of simple low-tech biogas digesters in water limited areas of SSA. With redesign of digesters to incorporate a larger inlet and outlet pipe, it is feasible to use undiluted fresh dung, which at 10.6% TS and 80.6% VS would produce 1.62 GJ of useful energy per cow per year, although amendments may be necessary for feedstocks with higher percentage TS or C:N ratios. Alternatively, current digester configurations with the addition of a simple filter system could be used to recycle water from a 50:50 dilution which would result in 1.41 GJ of useful energy per cow per year. Either method would reduce the need to collect water from potentially distant sources. Recycling filtered liquid digestate has the best effect on methane production and can reduce the water requirement by at least 75% of current practice. Both methods could adequately supply the energy demands of a rural family of at least five owning four head of cattle. Further research is required to investigate the potential inhibitory effects of VFA and ammoniacal-N concentrations when water is recycled in further phases.

## Supplementary Material

Supplementary Material

## Figures and Tables

**Table 1:** Operating conditions for the dilution experiments showing chemical oxygen demand (COD), total solids (TS), volatile solids (VS) and organic loading rate in terms of volatile solids (OLR_VS_) and chemical oxygen demand (OLR_COD_).

Reactor no.	Dung volume fed (mL/d)	Dung weight fed (g/d)	Water fed (mL/d)	TS fed (%)	TS fed (g/d)	VS fed (g/d)	COD of the feed (g/L)	OLR_VS_ (kg VS/ m^3^/d)	OLR_COD_ (kg COD/ m^3^/d)
V200	8.00	7.62	0	10.63	0.81	0.65	190.76	3.26	7.63
V133	5.33	5.07	2.67	7.08	0.54	0.44	127.17	2.17	5.08
V100	4.00	3.81	4.00	5.31	0.40	0.32	95.38	1.63	3.82
V67	2.67	2.54	5.33	3.54	0.27	0.22	63.59	1.09	2.54

d = day, water is deionised.

**Table 2:** Mean and standard error chemical oxygen demand (COD) and volatile solids (VS) reduction in dilution experiment.

Reactor group	% COD reduction, *P*_COD_R__		% VS reduction, *P*_VS_R__		COD removal, *R*_COD_R__ (g/L/day)		PH	
	Mean	SE	Mean	SE	Mean	SE	Mean	SE
V200	20.87	2.45	20.31	2.48	1.59	0.19	7.0	0.3
V133	7.59	3.07	12.25	1.48	0.39	0.16	6.5	0.2
V100	12.98	2.71	18.58	2.96	0.50	0.10	7.4	0.1
V67	6.23	7.77	17.23	6.19	0.16	0.20	7.5	0.2

**Table 3:** Properties of the feed used in the recycling experiments Note: Mean of 5–6 samples each (SE given in brackets). Dung: filtrate mixes based on theoretical values. COD = chemical oxygen demand, TS = total solids, VS = volatile solids.

Reactor phase	TS% RLD100	VS% RLD100	OLR VS kg/m^3^/d RLD100	COD g/L RLD100	TS% RLD50	VS% RLD50	ORL VS kg/m^3^/ d RLD50	COD g/L RLD50
Phase 1	7.02 (0.04)	5.44 (0.03)	1.99 (N/A)	121.73 (5.57)	6.17 (0.03)	4.86 (0.02)	1.84 (N/A)	108.56 (5.04)
Phase 2	7.58 (0.09)	5.98 (0.06)	2.25 (N/A)	122.71 (5.69)	6.45 (0.06)	5.13 (0.04)	1.75 (N/A)	109.05 (5.10)
Phase 3	7.46 (0.19)	5.85 (0.13)	2.18 (N/A)	123.34 (6.19)	6.39 (0.06)	5.06 (0.07)	1.87 (N/A)	109.36 (5.35)

d = day.

**Table 4:** Mean volatile solids (*P*_VS__R_) and chemical oxygen demand reduction (*P*_COD__R_) in the 3 recycling phases. Note: Mean values with standard error shown in brackets and significance refers to differences between phases

Reactors	Phase 1	Phase 2	Phase 3	Significant difference	P value	Whole run mean
Group RLD50% VS reduction	23.36 (0.85)	27.03 (1.06)	26.27 (0.69)	YES	0.02	25.61 (0.57)
Group RLD50% COD reduction	29.30 (4.00)	23.77 (1.36)	22.91 (2.21)	NO	0.77	24.53 (1.42)
Group RLD50 pH	7.50 (0.08)	7.64 (0.04)	7.46 (0.03)	YES	0.03	7.53 (0.03)
Group RLD100% VS reduction	29.14 (0.76)	31.52 (0.73)	29.25 (1.10)	NO	0.17	29.91 (0.56)
Group RLD100% COD reduction	36.02 (0.74)	27.94 (1.02)	25.03 (1.20)	YES	<0.01	28.26 (1.08)
Group RLD100 pH	7.58 (0.05)	7.65 (0.04)	7.58 (0.04)	NO	0.52	7.60 (0.02)

## Data Availability

Mendeley Data, doi: 10.17632/37f4dsgb4z.1 and doi:10.17632/mnwt9jyv6z.1.
