# Supplementary material for: Investigating the challenges of biogas provision in water limited environments through laboratory scale biodigesters
Source: Int J Sustain Energy. Author manuscript; Available in PMC 2023 Oct 9. (PMC7615168; doi:10.1080/14786451.2023.2235022)
Supplement: Supplementary Material [file EMS188508-supplement-Supplementary_Material.docx]

## Supplementary material for Mitigating the drawbacks of water availability for biogas production in sub-Saharan

### Supplementary Table 1: Summary of key figures used

| Component | Units | v200 | v133 | v100 | v67 | F100 | F50 |
| --- | --- | --- | --- | --- | --- | --- | --- |
| $P_{\mathrm{COD}_{R}}$ | % | 20.87 | 7.59 | 12.98 | 6.23 | 28.26 | 24.53 |
| ${COD}_{s}$ | g/L | 190.76 | 127.17 | 95.38 | 63.59 | 122.59 | 108.99 |
| $M_{\mathrm{dung}_{\mathrm{cow}}}$ | kg | 14 | 14 | 14 | 14 | 14 | 14 |
| $d_{\mathrm{dung}}$ | g/L | 952 | 952 | 952 | 952 | 952 | 952 |
| $R_{\mathrm{COD}_{R,cow}}$ | kg/cow/day | 0.59 | 0.14 | 0.18 | 0.06 | 0.51 | 0.39 |
| $R_{CH4,cow}$ | kg/cow/day | 0.15 | 0.04 | 0.05 | 0.01 | 0.13 | 0.10 |
| $HV$ | MJ/kg | 55 | 55 | 55 | 55 | 55 | 55 |
| $E day$ | MJ/cow/day | 8.05 | 1.95 | 2.50 | 0.80 | 7.01 | 5.41 |
| $E year$ | MJ/cow/year | 2938 | 712 | 914 | 292 | 2557 | 1973 |
| ${E year}_{useful}$ | MJ/cow/year | 1616 | 392 | 503 | 161 | 1406 | 1085 |

Where $P_{\mathrm{COD}_{R}}$is the percentage reduction of COD, ${COD}_{s}$ is the theoretical COD in the feed (g/L), based on untreated dung adjusted for dilution, $M_{\mathrm{dung}_{\mathrm{cow}}}$is the daily dung production rate per cow, $d_{\mathrm{dung}}$ is the density of the cow dung, $R_{\mathrm{COD}_{R,cow}}$is the COD reduction over the digestion period from the dung produced each day by one cow, $HV$ is the heating value of CH4, $E day$ is energy output per cow per day, $E year$ is energy output per cow per year, and ${E year}_{useful}$ is energy available from one cow for cooking, assuming 55% efficiency of biogas stoves.


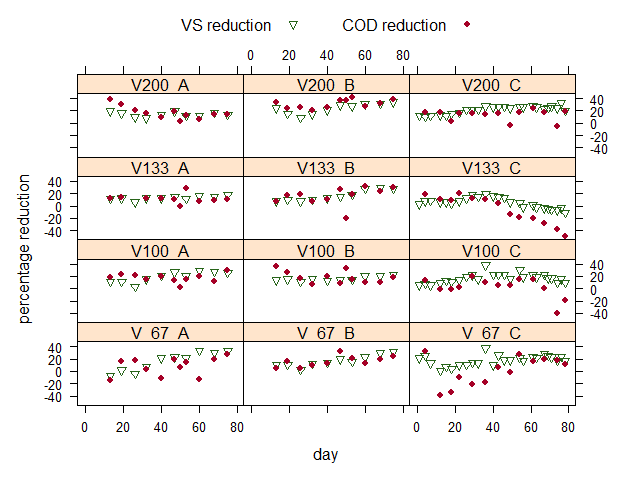
 Supplementary Figure 1: VS and COD reduction for individual reactors in dilution experiment


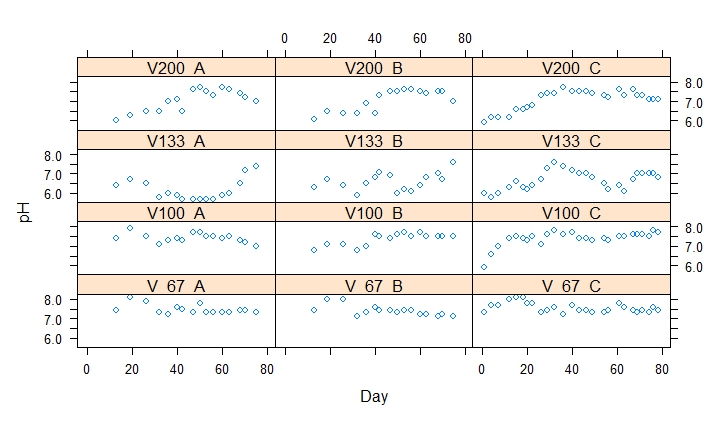


### Supplementary Figure 2: pH over running time for individual reactors in dilution experiment


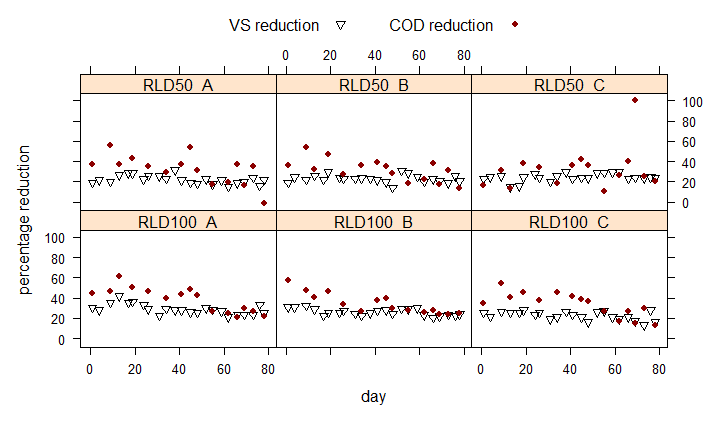


### Supplementary Figure 3: VS and COD reduction for individual recycled liquid digestate experiment


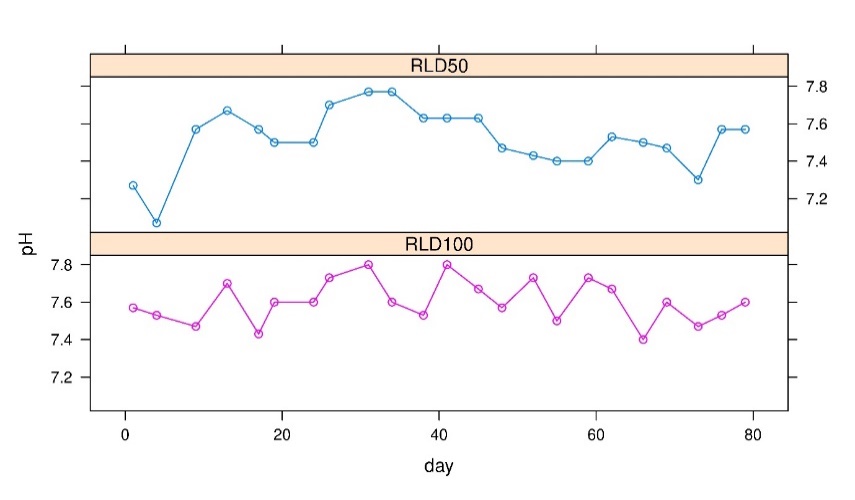


### Supplementary Figure 4: Mean pH over running time in recycled liquid digestate experiment


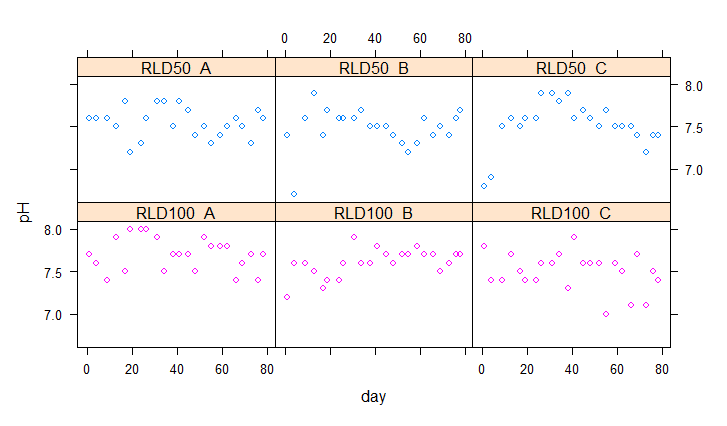


### Supplementary Figure 5: pH over running time for individual reactors in recycled liquid digestate experiment
